# Comparative Genomics of Disease and Carriage Serotype 1 Pneumococci

**DOI:** 10.1093/gbe/evac052

**Published:** 2022-04-19

**Authors:** Chrispin Chaguza, Chinelo Ebruke, Madikay Senghore, Stephanie W. Lo, Peggy-Estelle Tientcheu, Rebecca A. Gladstone, Gerry Tonkin-Hill, Jennifer E. Cornick, Marie Yang, Archibald Worwui, Lesley McGee, Robert F. Breiman, Keith P. Klugman, Aras Kadioglu, Dean B. Everett, Grant Mackenzie, Nicholas J. Croucher, Anna Roca, Brenda A. Kwambana-Adams, Martin Antonio, Stephen D. Bentley

**Affiliations:** 1 Parasites and Microbes Programme, Wellcome Sanger Institute, Wellcome Genome Campus, Cambridge, UK; 2 Darwin College, University of Cambridge, Silver Street, Cambridge, UK; 3 Department of Clinical Infection, Microbiology and Immunology, Institute of Infection, Veterinary and Ecological Sciences, University of Liverpool, Liverpool, UK; 4 Medical Research Council (MRC) Unit The Gambia at the London School of Hygiene and Tropical Medicine, Fajara, The Gambia; 5 Department of Epidemiology, Center for Communicable Disease Dynamics, Harvard T.H. Chan School of Public Health, Boston, MA, USA; 6 Department of Biostatistics, University of Oslo, Oslo, Norway; 7 Malawi-Liverpool-Wellcome Trust Clinical Research Programme, Blantyre, Malawi; 8 Respiratory Diseases Branch, Centers for Disease Control and Prevention, Atlanta, GA, USA; 9 Hubert Department of Global Health, Rollins School of Public Health, Emory University, Atlanta, GA, USA; 10 College of Medicine and Health Sciences, Khalifa University, Abu Dhabi, UAE; 11 Murdoch Children’s Research Institute, Parkville, Melbourne, VIC, Australia; 12 London School of Hygiene & Tropical Medicine, London, UK; 13 MRC Centre for Global Infectious Disease Analysis, Department of Infectious Disease Epidemiology, School of Public Health, Imperial College London, London, UK; 14 NIHR Global Health Research Unit on Mucosal Pathogens, Division of Infection and Immunity, University College London, London, UK; 15 Warwick Medical School, University of Warwick, Coventry, UK

**Keywords:** *Streptococcus pneumoniae*, genome-wide association study, bacterial genomics, genomic epidemiology, pathogenicity, invasiveness

## Abstract

The isolation of *Streptococcus pneumoniae* serotypes in systemic tissues of patients with invasive disease versus the nasopharynx of healthy individuals with asymptomatic carriage varies widely. Some serotypes are hyper-invasive, particularly serotype 1, but the underlying genetics remain poorly understood due to the rarity of carriage isolates, reducing the power of comparison with invasive isolates. Here, we use a well-controlled genome-wide association study to search for genetic variation associated with invasiveness of serotype 1 pneumococci from a serotype 1 endemic setting in Africa. We found no consensus evidence that certain genomic variation is overrepresented among isolates from patients with invasive disease than asymptomatic carriage. Overall, the genomic variation explained negligible phenotypic variability, suggesting a minimal effect on the disease status. Furthermore, changes in lineage distribution were seen with lineages replacing each other over time, highlighting the importance of continued pathogen surveillance. Our findings suggest that the hyper-invasiveness is an intrinsic property of the serotype 1 strains, not specific for a “disease-associated” subpopulation disproportionately harboring unique genomic variation.

**Significance**

*Streptococcus pneumoniae* serotype 1 strains are a significant endemic cause of invasive diseases globally, especially in sub-Saharan Africa but are rarely detected in asymptomatic carriers, raising questions regarding the genetic similarity between these carriage and disease-associated isolates. We sequenced the first extensive collection of carriage and disease serotype 1 isolates and conducted a bacterial genome-wide association study to identify potential pathogenicity loci in this hyper-invasive serotype. Our findings show no evidence for the presence of specific disease-associated strains enriched with genomic variation promoting invasion.

## Introduction

*Streptococcus pneumoniae*, also known as the pneumococcus, is an opportunistic human pathogen that asymptomatically colonizes the respiratory tract but sometimes causes life-threatening diseases, including pneumonia, bacteremia, and meningitis (Henriques-Normark and Tuomanen 2013). Despite the widespread use of serotype-specific capsule-based pneumococcal conjugate vaccines, the pneumococcus remains a significant cause of life-threatening diseases (Wahl et al. 2018). These diseases account for >320,000 deaths globally each year in children <5 years old, and two-thirds occur in sub-Saharan Africa with a disproportionate representation of the hypervirulent serotype 1 strains (Johnson et al. 2010). The serotypes discovered to date (≈100) (Ganaie et al. 2020) vary substantially in how they evolve (Chewapreecha, Harris, et al. 2014), asymptomatically colonize the nasopharynx (Abdullahi et al. 2012), and cause invasive diseases in humans (Brueggemann et al. 2003; Balsells et al. 2018). Although most serotypes are proficient colonizers with modest invasive potential, some serotypes, notably serotype 1, are hyper-invasive as indicated by high disease-to-carriage odds ratios (Brueggemann et al. 2003) and progression rates (Løchen et al. 2021). Whereas nasopharyngeal carriage surveys typically show carriage rates of <1% for serotype 1 strains (Ebruke et al. 2015; Usuf et al. 2015; Usuf et al. 2019), >20% of patients with invasive pneumococcal diseases, in endemic sub-Saharan African settings, are due to serotype 1 (Johnson et al. 2010; Everett et al. 2012; du Plessis et al. 2016), often associated with lethal meningitis outbreaks (Leimkugel et al. 2005; Antonio et al. 2008; Mehiri-Zghal et al. 2010; Kwambana-Adams et al. 2016; Franklin et al. 2021).

Considering the high pneumococcal nasopharyngeal carriage rates in sub-Saharan Africa (Hill et al. 2006; Ebruke et al. 2015; Usuf et al. 2019; Swarthout et al. 2020), the rarity of serotype 1 in the nasopharyngeal niche is puzzling given its frequency in disease (Ritchie et al. 2012). This infrequent carriage but high disease burden due to serotype 1 pneumococci seems to contradict the conventional assumption that carriage is necessary to develop invasive pneumococcal diseases (Bogaert et al. 2004; Simell et al. 2012). The basis for the hyper-invasiveness of serotype 1 pneumococci, reflecting rare and short carriage but high abundance in disease, remains poorly understood (Brueggemann et al. 2003; Abdullahi et al. 2012; Usuf et al. 2015). Whether the serotype 1 isolates sampled from patients with invasive diseases represent a unique subpopulation genetically distinct from those sampled in the nasopharyngeal niche is unknown, due to the rare isolation in asymptomatic individuals. To date, the majority of studies have focused on comparing serotype 1 clones with other serotypes (Bricio-Moreno et al. 2017; Jacques et al. 2020). Direct population-level comparison of serotype 1 isolates from invasive disease and asymptomatic carriage are urgently needed to provide insights regarding the pathogenicity of this hypervirulent serotype. However, the scarcity of serotype 1 carriage isolates makes the genetic comparison with the readily available disease-associated strains challenging.

The application of genome-wide association studies (GWAS) to identify novel genetic variants linked with bacterial phenotypes has increased over the past decade (Read and Massey 2014; Power et al. 2016). This agnostic approach does not require prior hypotheses about specific candidate loci; therefore, it is unbiased at detecting causal genetic variation even for incompletely studied phenotypes. Recent advances in whole-genome sequencing technologies and associated cost reductions have increased the applicability of GWAS. In parallel, advances in GWAS, such as the development of linear mixed models that robustly account for the clonal structure, have drastically improved genotype–phenotype associations in bacterial pathogens (Read and Massey 2014; Power et al. 2016). The application of bacterial GWAS has identified associations between genomic loci and phenotypes such as disease susceptibility (Young et al. 2019); duration of asymptomatic carriage (Lees, Croucher, et al. 2017); progression between tissues (Lees, Kremer, et al. 2017; Chaguza et al. 2020); virulence (Laabei et al. 2014); environmental and host adaptation (Ma et al. 2020); nutrient synthesis (Sheppard et al. 2013); and antimicrobial resistance (Chewapreecha, Marttinen, et al. 2014; Coll et al. 2018). Such application of GWAS to study pathogen biology has the potential to reveal pathogenicity loci, which could inform disease prevention and control measures.

Here we investigated genomic differences between pneumococcal serotype 1 isolates sampled from the nasopharynx of asymptomatic carriers in the community and clinical specimens collected from patients with invasive pneumococcal disease at the hospital in The Gambia (West Africa)—a setting with high pneumococcal carriage and disease burden (Roca et al. 2011). Using well-controlled GWAS approaches, we assessed whether the rarely detected asymptomatic serotype 1 carriage isolates disproportionately harbor unique genomic variation distinct from those causing diseases, which impedes their ability to cause invasive disease.

## Results

### Population Structure of Serotype 1 *S. pneumoniae*

**Fig. 1.**
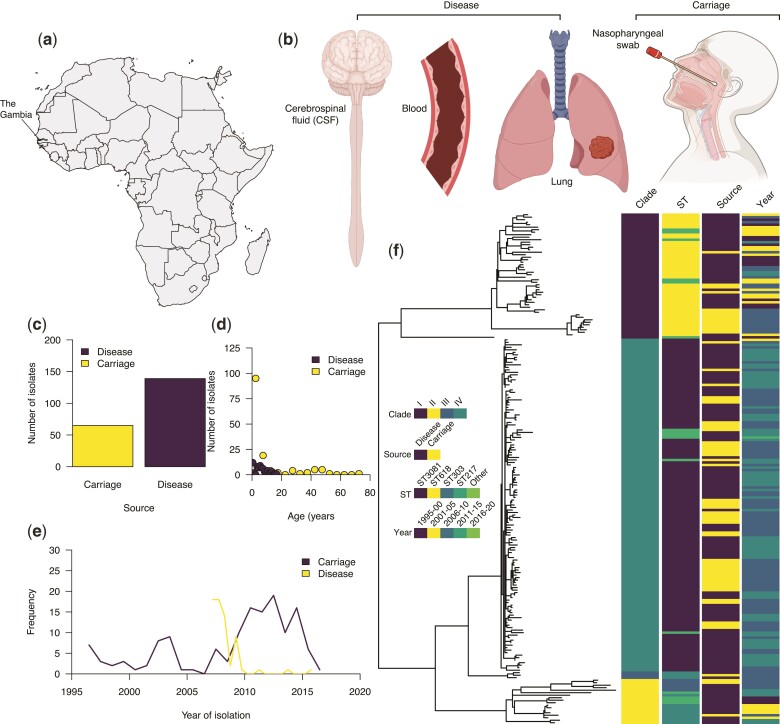
Characteristics and genetic relatedness of the pneumococcal serotype 1 isolates used in the study. (*a*) Map of Africa showing the location of The Gambia in West Africa where the study was conducted. (*b*) Disease status as defined based on the anatomical site of the human body showing where the serotype 1 isolates used in the study were sampled from. (*c*) Bar plot showing the number of whole-genome sequenced serotype 1 isolates from the carriage (*n* = 65) and disease (*n* = 139). (*d*) Distribution of the serotype 1 isolates from carriage and disease by the age of the individuals. (*e*) Line plot showing the temporal distribution of the serotype 1 isolates from carriage and disease. (*f*) A maximum-likelihood phylogenetic tree constructed after removing SNPs in recombinogenic regions showing genetic relatedness of the carriage and disease serotype 1 isolates. The icons in (*b*) shown in the figure were created with permission in BioRender.com (https://biorender.com/).

We analyzed the genomes of 204 serotype 1 isolates; 65 isolates from individuals with asymptomatic carriage and 139 from patients with invasive disease, collected from 1996 to 2016 in The Gambia, West Africa (fig. 1*a–e*, supplementary fig. 1 and data 1, Supplementary Material online). The isolates associated with invasive disease were sampled from different sources; blood (*n* = 94), cerebrospinal fluid (*n* = 15), lung aspirates (*n* = 27), pleural aspirates (*n* = 2), and pus (*n* = 1). All the isolates were assigned to the global pneumococcal sequence cluster 2 (GPSC2) lineage based on the global pneumococcal sequencing (GPS) nomenclature (Gladstone et al. 2019). In terms of multilocus sequence typing (MLST) (Brueggemann and Spratt 2003), GPSC2 corresponds to the clonal complex 217 (CC217), predominantly found in sub-Saharan Africa (Brueggemann and Spratt 2003).

We constructed a maximum-likelihood phylogeny of the isolates using genomes with single nucleotide polymorphisms (SNPs) located in regions containing putative recombination events excluded. The phylogeny revealed four clades for the isolates included in this study designated as clades I–IV (fig. 1*f*). Clades I and II were associated with the fewest isolates, mainly belonging to ST217 and ST303. Clade IV, which is predominantly associated with ST3081, replaced ST618 strains in clade I in the mid-2000s (Ebruke et al. 2015). We next assessed the genomic variation in the capsule biosynthesis locus by generating a maximum-likelihood phylogeny of the isolates. The isolates were placed into two sequence clusters of ST618-like and ST217 or ST3081-like strains, corresponding to the main STs identified in The Gambia. These clustering patterns were consistent with those seen in the whole-genome phylogeny of the serotype 1 isolates (supplementary fig. 2, Supplementary Material online). To quantify the effect of the pneumococcal genetic background on the disease status of the isolates, we used a generalized linear regression model to investigate the association between the clades and disease status. We defined the disease status as isolation of the pneumococcus from the nasopharynx of asymptomatic carriers or systemic tissues of patients with invasive diseases. The distribution of carriage and disease isolates varied across the phylogenetic tree possibly highlighting the differences in the sampling of isolates across clades or the invasiveness of the strains across distinct genetic backgrounds. Such variability indicated that controlling for the population structure was necessary for the GWAS to identify genetic variants associated with disease status.

### A Strong Correlation between Phylogeny and Disease Status

**Fig. 2.**
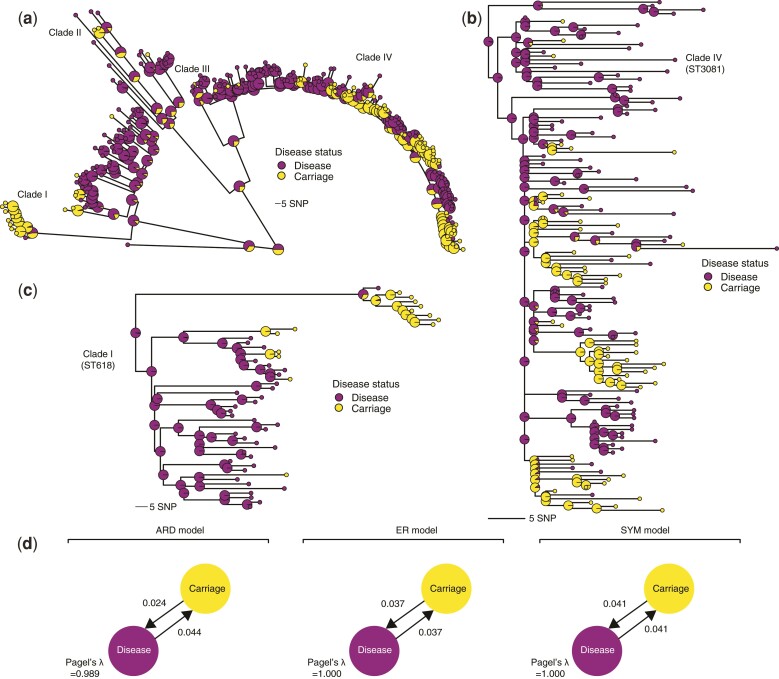
Phylogenetic signal and distribution of the pneumococcal serotype 1 isolates based on disease status. (*a*) Maximum-likelihood phylogeny showing the posterior probability of each disease status states at each terminal and internal node of the tree based on stochastic ancestral character reconstruction. The internal nodes are drawn with a larger radius to distinguish them from the terminal nodes. The colors of the nodes represent the disease status of the isolates as shown in the key next to the phylogenetic tree. (*b*) Phylogenetic tree of a subset of the serotype 1 isolates belonging to clade IV, which is predominantly associated with ST3081, the most common serotype 1 ST in The Gambia, West Africa. (*c*) The zoomed-in phylogenetic tree of the isolates belonging to clade I containing isolates belonging to ST618, which was the most dominant serotype 1 ST in The Gambia before its replacement by ST3081 in the early 2000s. (*d*) Estimated genetic signals associated with disease status of the serotype 1 isolates using the Pagel’s *λ* statistic. The transition rates between disease states are shown next to the arrows and the values of the Pagel’s *λ* statistic are shown at the bottom of the diagram for each model.

We first assessed the correlation between phylogeny and disease status using Pagel’s *λ* statistic (Pagel 1999) (fig. 2*a–c*). Three discrete character models (all rates different [ARD], equal rates[ER], and symmetric [SYM] model) were used to infer Pagel’s *λ* values which range from 0 to 1 with high values indicating the presence of a strong phylogenetic signal. We observed similar mean estimates for Pagel’s *λ* after subsampling the phylogenetic tree to an equal number of isolates for each disease status trait to minimize sampling biases. The inferred Pagel’s *λ* values for the ARD, ER, and SYM models were 0.98, 1.00, and 1.00, respectively, which suggested that the disease status phenotype evolved rapidly and strongly correlated with the phylogenetic tree (fig. 2*d*). Such correlated distribution of the disease status with the phylogeny suggested a robust phylogenetic signal, potentially suggesting that there may be distinguishable clusters of isolates associated with disease status. This implied that specific genomic variation associated with these clusters of isolates may influence the invasiveness of the pneumococcal serotype 1 isolates.

### Multiple GWAS Methods to Link Genomic Variation with Disease Status

**Fig. 3.**
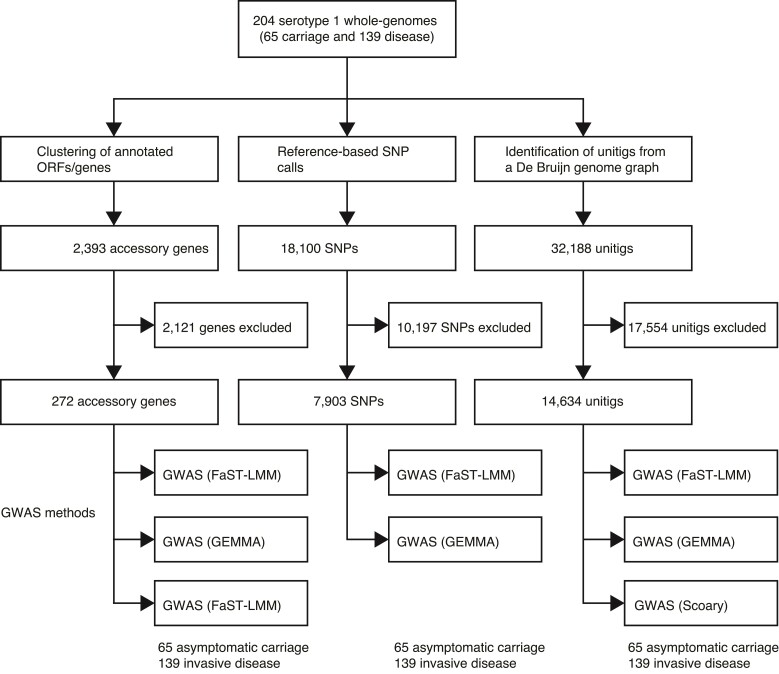
Overview of the GWASs performed in this study using different methods and types of genetic variation. Summary of the number of pneumococcal serotype 1 isolates sampled from healthy individuals with asymptomatic carriage and patients with invasive diseases. Three different types of genetic variation, namely, presence/absence of accessory genes, SNPs, and unitigs, were used for the GWAS. Each type of genetic variation was analyzed using multiple approaches, two linear mixed model methods (FaST-LMM and GEMMA) and phylogenetic or evolutionary convergence-based method (Scoary).

We next performed a GWAS using a linear mixed model to identify genetic determinants associated with the disease status (disease or carriage) of the isolates shown in figure 1*f*. To avoid inherent limitations of individual GWAS methods, we used two linear mixed models (FaST-LMM, Lippert et al. 2011 and GEMMA, Zhou and Stephens 2012) and a phylogenetic- or convergence-based approach, Scoary (Brynildsrud et al. 2016) (fig. 3). We focused on genetic variation consistently showing adjusted *P-*value < 0.05 using the three GWAS methods to triangulate potential hits, whereas minimizing false-positive associations. To control potential confounders in the GWAS, including population structure explicitly as a random effect (GEMMA and FaST-LMM) and implicitly using the phylogenetic tree (Scoary), and sequence read length, and an individuals’ age as a fixed covariate. To control for potential batch effects due to different sequence read lengths, we trimmed the longer reads to the same length and reassembled the genomes before the GWAS. Because GWAS using SNPs, genes, and unitigs have inherent limitations, we used all the three variant types to guard against shortfalls pertinent to each data type. The quantile–quantile (QQ) plots to compare the observed and expected *P-*values revealed no apparent issues, reflecting adequate control for the population structure of the isolates as a significant confounder in bacterial GWAS (supplementary fig. 3, Supplementary Material online).

GWAS based on 7,903 out of 15,411 SNPs, which passed the filtering based on the minor allele frequency and absence, revealed no consensus statistically significant associations with the disease status based on both FaST-LMM and GEMMA (supplementary fig. 4, Supplementary Material online). SNPs capture single nucleotide substitutions based only on the information available in the reference genome but not insertions or deletions. To address this, we performed an additional GWAS analysis using the presence and absence patterns of unitig sequences identified in the entire dataset. Unlike SNPs, unitig sequences effectively capture allelic variants at different resolutions, including SNPs, insertion, and deletions in coding and noncoding regions, and genomic rearrangements.

**Fig. 4.**
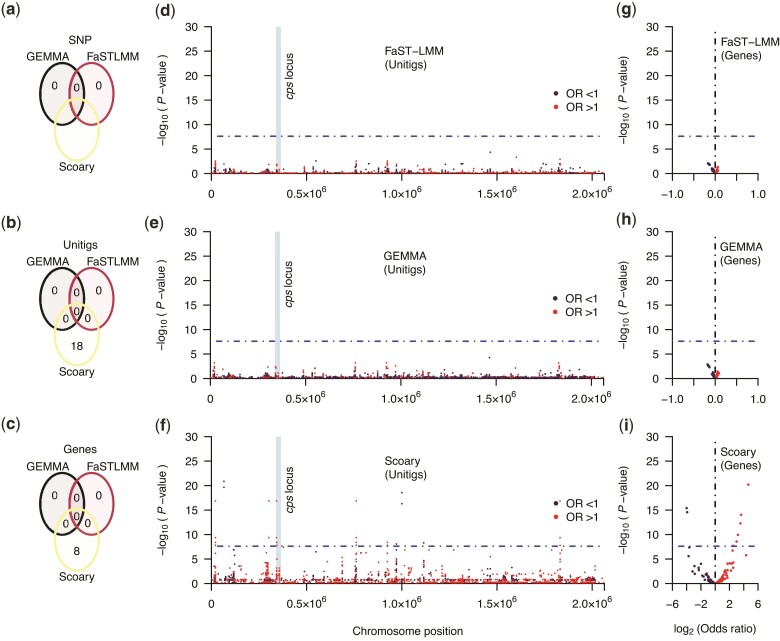
Manhattan and volcano plots showing statistical significance and effect sizes of the genetic variants associated with disease status in the GWAS. Venn diagrams showing the number of statistically significant (*a*) SNPs, (*b*) accessory genes, and (*c*) unitig sequences identified by each GWAS method. The total number of variants tested is specified in the title for *a*–*c*. The absence data for Scoary in (*a*) reflect the fact that we did not run GWAS of the SNPs using this method. Manhattan plots showing statistical significance (-log_10_[unadjusted *P-*value]) and chromosomal location of the unitig sequences for the GWAS using (*d*) FaST-LMM, (*e*) GEMMA, and (*f*) Scoary. Volcano plot showing the relationship between statistical significance (-log_10_[unadjusted *P-*value]) and the effect size in terms of the log-transformed (base 2) asymptomatic carriage-to-disease odds ratio for the accessory gene sequences for the GWAS using (*g*) FaST-LMM, (*h*) GEMMA, and (*i*) Scoary. The points in all the graphs are colored based on the odds ratio, as shown in the key on the right of each diagram. The blue line represents the genome-wide statistical significance threshold based on the Bonferroni adjustment. The unitig sequences shown in *g*–*i* were mapped to a complete reference genome for serotype 1 strain PNI0373 from The Gambia belonging to sequence type ST168 (GenBank accession: CP001845).

Complementary GWAS based on the final set of 14,634 unitig sequences (out of 32,188) revealed no consensus statistically significant associations based on the three GWAS approaches (fig. 4*d–f*, supplementary data 2, Supplementary Material online). Specifically, FaST-LMM and GEMMA found no statistically significant associations, whereas Scoary identified 18 statistically significant associations. The unitig sequences identified by Scoary were annotated by comparing them to pneumococcal reference genomes. Of the annotated unitig sequences, ≈50% were associated with transposase or insertion sequences, whereas the rest were either in intergenic regions (≈28%) or their annotations were not available (≈22%). These insertion sequences appear to be widespread across the genomes driven by duplication events. Interestingly, no unitig sequences were statistically associated with the disease status mapped to the genomic sequences within the capsule biosynthesis locus (fig. 4*d–f*, supplementary fig. 5, Supplementary Material online). The absence of support by the other tools such as FaST-LMM and GEMMA suggested that further validation of the findings is required to confirm the impact of these genomic variations on the invasiveness of serotype 1 pneumococci.

We then undertook a complementary GWAS to assess whether the presence and absence of accessory genes, regardless of any mutations within them, were associated with the disease status (fig. 3). The pan-genome size of the serotype 1 isolates comprised of 2,393 genes, of which 292 present between 5% and 95% of the isolates were used for the GWAS. Consistent with the results from the GWAS using SNPs and unitigs, we found no consensus statistically significant associations using the three GWAS methods (fig. 4*g–i*, supplementary data 2, Supplementary Material online). However, although GEMMA and FaST-LMM identified no statistically significant associations, Scoary identified four genes statistically associated with disease status. Consistent with the findings from Scoary using unitig sequences, most of the statistically significant genes (≈75%) were associated with transposase and insertion sequences (fig. 4*a–f*). These insertion sequences were highly conserved genetically but with paralogs distributed across the genomes. The statistically significant associations identified by Scoary suggested that genomic variation tagging these insertion sequences may influence the invasiveness of pneumococcal serotype 1. However, validation of the associations identified by Scoary is required as the results were inconsistent with the linear mixed model GWAS approaches.

### Minimal Contribution of Genomic Variation on Disease Status

To quantify the amount of the variability in the phenotype, that is, disease status, explained by genetics, we estimated the narrow-sense heritability. Because the distribution of genomic variants varies by genomic background and may be influenced by other factors, we also controlled for the population structure and other covariates as done for the GWAS. We found a narrow-sense heritability of ∼0, suggesting a negligible influence of pneumococcal genomic variation on the invasiveness of serotype 1 pneumococci. Such a low estimate was consistent with the GWAS results showing the absence of consensus genomic variation statistically associated with disease status.

## Discussion

Although the hyper-invasiveness and virulence of pneumococcal serotype 1 strains is well known (Brueggemann et al. 2003), the genetic basis for their invasiveness remains poorly understood. In this study, we compared serotype 1 isolates sampled from patients with invasive diseases and asymptomatic carriage using GWAS approaches to determine whether the presence or absence of certain genomic variations, within isolates of the same serotype, enhance or impede invasiveness. Our findings show that serotype 1 isolates sampled from healthy individuals and patients with invasive diseases are not genetically distinct, suggesting that strains sampled from patients with invasive diseases do not represent a subpopulation containing unique genetic variation associated with greater invasiveness than their carriage counterparts, and vice versa. These findings support the notion that the outer cell wall polysaccharide capsule, the main pneumococcal virulence factor (Kadioglu et al. 2008), is the primary determinant of the hyper-invasiveness of serotype 1 strains.

The absence of consensus on statistically significant associations between genomic variation in serotype 1 isolates and disease status supports the opportunistic infection model, whereby serotype 1 pneumococci isolated from carriage and disease are equally able to cause disease (Méric et al. 2018). In these opportunistic pathogens, disease-causing strains are distributed across multiple genetic backgrounds rather than restricted to specific clones in the phylogeny. Our findings suggest that the hyper-invasiveness of serotype 1 strains is an intrinsic property shared by all serotype 1 pneumococci regardless of whether they are found in patients with diseases or asymptomatic carriage. However, although we did not find consensus genomic loci associated with invasion, there was a strong phylogenetic signal for disease status suggesting that some strains or clades are highly correlated with the disease state. These findings implied that strain or lineage effects may also be an important determinant of whether colonization with serotype 1 pneumococci evolves into disease. It remains to be seen whether such strain differences are driven by the capsule as the phylogeny of the capsule biosynthesis region revealed clusters of the ST618-like and ST217/ST3081-like strains consistent with the whole-genome phylogeny. Why serotype 1 is rare during carriage compared with other serotypes remains an open question to be addressed by further studies. However, due to the suspected low levels of recombination of serotype 1 strains (Chaguza et al. 2016), it is possible that these strains are inefficient colonizers as they cannot easily adapt to environmental changes.

Although the three GWAS methods identified no consensus genomic variants, Scoary identified statistically significant associations. Whether these variants are biologically plausible and not merely analysis artifacts remain to be determined. Because Scoary infers genotype–phenotype associations based on phylogenetic convergence of the genotype and phenotypes, it may have a higher sensitivity, especially for clonal populations, than the classical GWAS approaches (Brynildsrud et al. 2016). However, this may come with an increase in the false-positive rate, as Scoary only implicitly controls for population structure, but not for other covariates. Assuming the statistically significant associations identified by Scoary are not false-positives, certain genomic variation mostly associated with IS elements is overrepresented in the carriage than disease isolates, negatively impacting the invasiveness of serotype 1 strains. This finding is consistent with previous studies which showed the role of mobile genetic elements on pathogenicity, virulence, and adaptation of other bacterial species, including *Staphylococcus epidermidis* (IS*256*) (Both et al. 2021), *Mycobacterium bovis* (IS*6110*) (Soto et al. 2004), *Neisseria meningitidis* (IS*1301*) (Uria et al. 2008), *Escherichia coli* (IS*3*) (Aronson et al. 1989) and *Xanthobacter autotrophicus* (IS*1247*) (van der Ploeg et al. 1995). Potentially, as the insertion sequences replicate and increase in numbers in a genome of the less invasive strains, they may exert a fitness effect that makes the strain less able to cause disease, confining them to a nasopharyngeal carriage lifestyle, that is, impeding invasion but not necessarily enhancing the ability to colonize. However, there could be a cyclical effect where the insertion sequences are sometimes purged from the genome, allowing the fitter strain to invade. Because the associations were not consensus, that is, inferred by a single GWAS approach only, further studies are required to validate or rule out the biological plausibility of these findings. Nevertheless, our study demonstrates that the findings from different GWAS tools are not always consistent; therefore, it is crucial to select the most appropriate GWAS methods for specific datasets, for example, based on the clonality of the isolates, and to adjust for potential confounders. Overall, we recommend using multiple GWAS methods and report consensus genomic variation identified by all or most of the tools, when there is no objective rationale for using a specific approach, as highlighted in this study.

Our approach of sampling carriage serotype 1 isolates from asymptomatic individuals in the communities, and disease isolates from patients, eliminated ambiguity when defining the disease status of the isolates. By collecting the carriage isolates from asymptomatic individuals and not patients, we minimized the chances of sampling serotype 1 isolates transitioning to causing invasive disease, likely enhancing the statistical power to detect genetic signals associated with disease status. We also explicitly adjusted for the individuals’ age, sampling time, and population structure as these variables are well-known confounders in population-level epidemiological studies (Power et al. 2016). Because no GWAS method is perfect, we performed GWAS using multiple methods and forms of genetic variation to identify consensus genetic variation consistently found by different approaches to circumvent each method’s inherent limitations and biases, thereby minimizing false-positive hits. However, there are some limitations to be taken into consideration. First, our dataset size may not be seen as significant compared with datasets used in previously reported bacterial GWAS studies (Li et al. 2019), but in the context of pneumococcal serotype 1, it represents a unique and large dataset of carriage isolates to date, which required extensive and costly nasopharyngeal carriage surveys in a resource-limited setting to amass sufficient samples due to the rarity of this serotype in asymptomatic carriers. Future GWAS studies should conduct power calculations to determine the required sample sizes rather than using convenient samples as done in our study to improve the power to detect potential statistically significant differences in the GWAS. Second, not all the isolates were sequenced using the same read length; therefore, we cannot rule out potential batch effects in the GWAS (Young et al. 2021) and other next-generation sequencing datasets (Leigh et al. 2018). We trimmed the longer reads (125 bp) to the same length (100 bp) and included read length as a covariate in the analysis to account for such potential batch effects. Third, although we corrected for the variability in the time of isolation and age of the individuals, we did not explicitly correct for the geographical area. However, the effect of the variability in the geographical area would be minimal as most of the individuals came from the same communities. Fourth, we did not replicate our GWAS using an external validation dataset because there are no similar datasets of carriage and disease serotype 1 pneumococci. However, the availability of such datasets in the future will allow for studies to validate our findings, including assessing differences between disparate geographical settings.

By undertaking extensive carriage surveys and hospital surveillance to amass a unique collection of pneumococcal serotype 1 isolates, we have found no consensus evidence of genomic variation distinguishing isolates associated with asymptomatic carriage and invasive disease. These findings suggest that serotype 1 strains are intrinsically hyper-invasive and equally likely to cause disease; there are no consistent loci more commonly associated with invasive diseases than carriage, and vice versa. Our study represents the first comprehensive comparative genomic analysis of carriage and disease isolates to understand the impact of genomic variation on the pathogenicity of serotype 1 strains. However, much remains to be done to validate these findings using additional geographically diverse datasets and to experimentally assess whether the nonconsensus statistically significant associations in the insertion sequences identified by some GWAS approaches are not merely artifacts. With the increasing availability of large and well-sampled pneumococcal genomic datasets globally, the application of GWAS and other computational methods could unpick cryptic genotype–phenotype associations not detected in this study, potentially unlocking novel mechanisms of pathogenicity, adaptation, and transmission.

Our study highlights the utility of genomic surveillance and genotype–phenotype association studies to provide novel, unbiased, hypothesis-free, and genome-wide insights into the population-level pathogen traits, such as invasiveness, which is intrinsically challenging to study experimentally, to inform infection prevention and control strategies.

## Materials and Methods

### Sample Characteristics and Preparation

We selected 204 pneumococcal serotype 1 isolates for whole-genome sequencing in The Gambia, West Africa (supplementary data 1, Supplementary Material online). Of these isolates, 139 were sampled from the clinical specimens of patients with invasive diseases between 1996 and 2016, whereas 65 were isolated from the nasopharynx of asymptomatic individuals. The disease-associated isolates were collected at the Medical Research Council (MRC) clinic in Fajara and Basse, but some patients were referred from other, primarily teaching, hospitals. Hence, the samples represent the greater Banjul area (Western Region) and Basse (Upper River Region). The carriage isolates were recovered from multiple studies between 2007 and 2016, with the majority of the isolates sampled between 2007 and 2009 via different studies, including a large-scale carriage survey of >12,000 people as described previously (Ebruke et al. 2015). Based on this survey, the prevalence of pneumococcus among carriers, determined using latex agglutination, was 71.78% in the pre-PCV7 period and 47.08% in the post-PCV7 period (Ebruke et al. 2015). Genomic DNA was extracted from fresh overnight cultures as described previously (Roca et al. 2015). All isolates included in the study were not associated with known pneumococcal serotype 1 outbreaks. The study protocols were approved by the MRC/Gambia Government Joint Ethics committee. We obtained informed consent from the participants or their parents or legal guardians before enrollment in the study.

### Quality Control, Assembly, and Annotation

Whole-genome sequencing was done using Illumina sequencing platforms (Illumina, CA, USA) through the Pneumococcal African Genomics (Cornick et al. 2015) and GPS consortium projects (https://www.pneumogen.net/gps/) (Gladstone et al. 2019). Quality control was undertaken to assess abnormalities in the sequence data. We included sequences with >60% reads mapping to *S. pneumoniae* using Kraken (version 2.1.2) (Wood and Salzberg 2014), >20× overall sequencing depth and >60% mapping coverage against *S. pneumoniae* ATCC 700669 reference genome (GenBank accession: NC_011900). We also excluded samples with >15% of the total heterozygous SNPs, and maximum minor allele frequency >25%, which was suggestive of a mixed sample. Furthermore, only draft genome assemblies with a total number of nucleotide bases between 2.0 and 2.2 Mb, consistent with the known genome size of the pneumococcus (Tettelin et al. 2001), were included in the analysis. No genomes were excluded from the analysis after the quality control. To minimize batch effects arising in the GWAS arising due to differences in the read lengths, we trimmed longer reads of 125–100 bp, to be consistent with the rest of the reads, by clipping an equal number of nucleotides at the 5′ and 3′ ends of the reads using seqtk (version 1.3-r117-dirty) (Shen et al. 2016). Genome assembly was done using SPAdes genome assembler (version 3.14.0) (Bankevich et al. 2012) and assembly statistics were generated using assembly-stats (version 1.0.1) (https://github.com/sanger-pathogens/assembly-stats). The mean genome size was 2,053,649 bp (range: 2,029,496–2,132,262 bp) and number of contigs was 253 (range: 161–527) (supplementary data 1, Supplementary Material online).

### Determination of Serotypes and Sequence Types

The isolates were serotyped using an in silico genomic serotyping approach implemented in SeroBA (version 1.0.0) (Epping et al. 2018). The isolates were initially serotyped using latex agglutination as previously described by Ebruke et al. (2015), therefore, the isolates expressed the capsule. Pneumococcal sequence types defined by the pneumococcal MLST scheme (Enright and Spratt 1998) were called using MLSTcheck (version 2.0.1510612) (Page, Taylor, Keane, 2016). We assigned each isolate to a pneumococcal lineage using PopPUNK (version 1.1.7) (Lees et al. 2019) based on the GPSC nomenclature defined by the GPS project (Gladstone et al. 2019).

### Population Structure and Phylogenetic Analysis

A multi-sequence whole-genome alignment was generated based on consensus sequences of each isolate inferred after mapping reads against a complete reference genome for serotype 1 strain PNI0373 from The Gambia (GenBank accession: CP001845) using Snippy (version 4.6.0) with default options (https://github.com/tseemann/snippy). We identified and extracted genomic positions from the alignment containing 18,100 SNPs in multi-FASTA and variant call format (VCF) using SNP-sites (version 2.3.2) (Page, Taylor, Delaney et al. 2016). The SNPs were used for clustering analysis to assign isolates into clades using the baps optimization option in Fastbaps (version 1.0.0) (Tonkin-Hill et al. 2019). For the phylogenetic construction, the SNPs located within putative recombination events were identified and excluded from the whole-genome alignment using Gubbins (version 1.4.10) (Croucher et al. 2015). A maximum-likelihood phylogenetic tree of the isolates was generated from the recombination-filtered alignment using RAxML (version 7.0.4) (Stamatakis 2006) with the GTR and Gamma model (Tavaré 1986, Yang 1993). The phylogenetic tree was rooted using an outgroup serotype 1 strain belonging to GPSC31 (sequence type ST306) predominantly found outside Africa (ENA accession: ERS628764). The inferred phylogeny was visually processed and explored using APE (version 4.3) (Paradis et al. 2004), and then annotated with isolate metadata using the “phylo4d” and “gridplot” functions in phylobase (version 0.8.6) (https://cran.r-project.org/package=phylobase), and phylosignal (version 1.3) packages, respectively (Keck et al. 2016). Stochastic discrete ancestral character reconstruction was used to map the disease status across the phylogenetic tree using the “ace” and “fitDiscrete” functions in the R packages APE (version 4.3) (Paradis et al. 2004) and Geiger (version 2.0.6.4), respectively (Pennell et al. 2014). The number of transitions between disease and carriage states and gain and loss of genetic variants, namely genes and unitig sequences, were inferred using “make.simmap” and “densityMap” functions in phytools (version 0.7.70) (Revell 2012). The difference in the mean number of transitions between states was assessed using the Kruskal–Wallis test. Correlation between the phylogenetic tree and the phenotype, or phylogenetic signal, was quantified using Pagel’s *λ* statistic (Pagel 1999).

### Detection of SNP, Accessory Gene, and Unitig Sequences

We generated input pedigree-formatted files for the GWAS from the VCF file of all SNPs identified in the whole-genome alignment of all the isolates using VCFtools (version 0.1.16) (Danecek et al. 2011). At genomic positions with >2 alleles, we generated biallelic variants using the two most common nucleotides. We then filtered out SNPs with minor allele frequency <5% or missingness >5% using PLINK (version 1.90b4) (Purcell et al. 2007). We used the two most common nucleotides detected at each position variants at each chromosomal position for the analysis. The coding sequences were identified in the draft genomes and annotated using Prokka (version 1.11) (Seemann 2014). The identified coding sequences were clustered to generate a matrix containing the presence and absence patterns of the clusters of orthologous genes (COGs) using the moderate stringency option in Panaroo (version 1.2.2) (Tonkin-Hill et al. 2020). We defined a core and accessory genes, were defined as COGs present in >99% and ≤99% of the isolates, respectively. The gene presence and absence data were merged with the phenotypic data to generate input pedigree-formatted files for the GWAS. Similarly, genes with minor allele frequency <5% were filtered out using PLINK (version 1.90b4) (Purcell et al. 2007). We then generated unitig sequences represented by nonbranching paths in the compacted De Bruijn graph constructed based on 31 bp *k*-mers from all the genomes using Bifrost (version 1.0.1) (Holley and Melsted 2020). We then queried all the unitig sequences inferred from the compacted De Bruijn graph of the entire dataset against the compacted De Bruijn graph to generate the presence and absence pattern of the unitig sequences in the isolates using Bifrost (version 1.0.1) (Holley and Melsted 2020). The ratio of *k*-mers from the queries present in the graph was specified as 1 when inferring the presence and absence patterns of the unitig sequence. The presence and absence matrix for the unitig sequences and the disease status phenotype were converted to input pedigree-formatted files for the GWAS and then processed to filter out unitig sequences with minor allele frequency <5% using PLINK.

### GWAS Analysis of SNPs, Accessory Genes, and Unitig Sequences

To assess the association between the genotype, that is, SNPs, and the unitig and accessory gene sequence presence and absence patterns, and phenotype, that is, disease status (carriage or disease), we performed univariate GWAS using linear mixed model methods, which accounts for the clonal population structure, in FaST-LMM (FastLmmC, version 2.07.20140723) (Lippert et al. 2011) and GEMMA (version 0.98.1) (Zhou and Stephens 2012). We treated the carriage isolates as controls and disease isolates as the affection status in the GWAS. The choice of the affection status has no impact on the statistical significance, although flipping it alters the direction but not the magnitude of the odds ratios or effect sizes. We coded the variants data as haploid human mitochondrial genotypes, designated as chromosome 26 as similarly done in bacterial GWAS analyses elsewhere (Chewapreecha, Marttinen, et al. 2014; Li et al. 2019). We also included age as a fixed covariate in the GWAS as it may influence the disease susceptibility of the individuals. We also undertook a complementary GWAS using a phylogenetic- or convergence-based method accounting for the clonal bacterial population structure with relaxed evolutionary assumptions implemented in Scoary (version 1.6.16) (Brynildsrud et al. 2016). The presence and absence matrices based on accessory gene and unitig sequences, encoded as 0 and 1, were converted into a Scoary-compatible format using a Python script developed by Dr. Jason Sahl (https://raw.githubusercontent.com/jasonsahl/LS-BSR/master/tools/BSR_to_scoary.py). For the single-locus linear mixed model GWAS, the population structure was calculated as a genetic relatedness matrix based on SNPs using “-gk 1” and “-fileSim” options in GEMMA and FaST-LMM, respectively. All the variants with adjusted *P*-value (*Q*-value) <0.05 based on the Bonferroni correction to control the false discovery rate due to multiple testing were reported as statistically significant. We used the genome size of serotype 1 strain gamPNI0373 (GenBank accession: CP001845), that is, 2,064,154 bp, as the number of possible independent variants to adjust the *P-*values. We used a “stringent” threshold of 0.05 divided by genome size to consider the genetic variants analyzed in the GWAS as statistically significant. The proportion of variance in the phenotype as explained by the pathogen genetics or heritability (*h*^2^) was estimated using GEMMA.

### Annotation of Genomic Variants

To annotate the identified unitig sequences, we compared each sequence to complete *S. pneumoniae* reference genomes obtained from GenBank using nucleotide BLAST (BLASTN) (version 2.5.0+) (Altschul et al. 1997). The presence of the unitig sequence in the genome was confirmed when the percent identity and coverage were >90%. The location of unitig sequences in the reference genomes was annotated using BLASTN and visually checked with ACT (version 9.0.5) (Carver et al. 2005). Venn diagrams were generated using “vennCounts” and “vennDiagram” function in limma (version 3.46.0) (Ritchie et al. 2015). Other statistical analyses were done using R (version 3.5.3) (R Core Team, 2020, http://www.R-project.org).

## Supplementary Material

evac052_Supplementary_Data

## Data Availability

The sequence data used in this study were deposited in the European Nucleotide Archive (ENA) and the accession numbers, isolate information and other source data are provided in supplementary data 1, Supplementary Material online. Other data used in this study, including the SNPs, accessory genes and unitig sequences, output data from the GWAS and the SPAdes genome assemblies generated from the trimmed sequencing reads, are publicly available online at https://github.com/ChrispinChaguza/Serotype1_Carriage_Disease_GWAS.
