# Supplementary material for: Comparative Genomics of Disease and Carriage Serotype 1 Pneumococci
Source: Genome Biol Evol. 2022 Apr 19;14(4):evac052. doi: 10.1093/gbe/evac052 (PMC9048925; doi:10.1093/gbe/evac052)
Supplement: evac052_Supplementary_Data [file evac052_supplementary_data.zip › Supplementary_information.docx]

**Comparative genomics of disease and carriage serotype 1 pneumococci**

Chrispin Chaguza, Chinelo Ebruke, Madikay Senghore, Stephanie W. Lo, Peggy-Estelle Tientcheu, Rebecca A. Gladstone, Jennifer E. Cornick, Gerry Tonkin-Hill, Marie Yang, Archibald Worwui, Catherine Turlan, Lesley McGee, Robert F. Breiman, Keith P. Klugman, Aras Kadioglu, Dean B. Everett, Grant Mackenzie, Nicholas J. Croucher, Anna Roca, Brenda A. Kwambana-Adams, Martin Antonio & Stephen D. Bentley

Other supplementary materials for this manuscript provided as separate files are as follows:

**Supplementary Data 1**: Summary of the carriage and disease pneumococcal serotype 1 isolates analysed in this study.

**Supplementary Data 2**: Summary of the statistically significant SNPs associated with disease status identified in the GWAS using Scoary. No statistically significant SNPs, genes and unitigs were identified using GEMMA and FaSTLMM. The GWAS using Scoary was done based on the genes and unitigs but not SNPs.


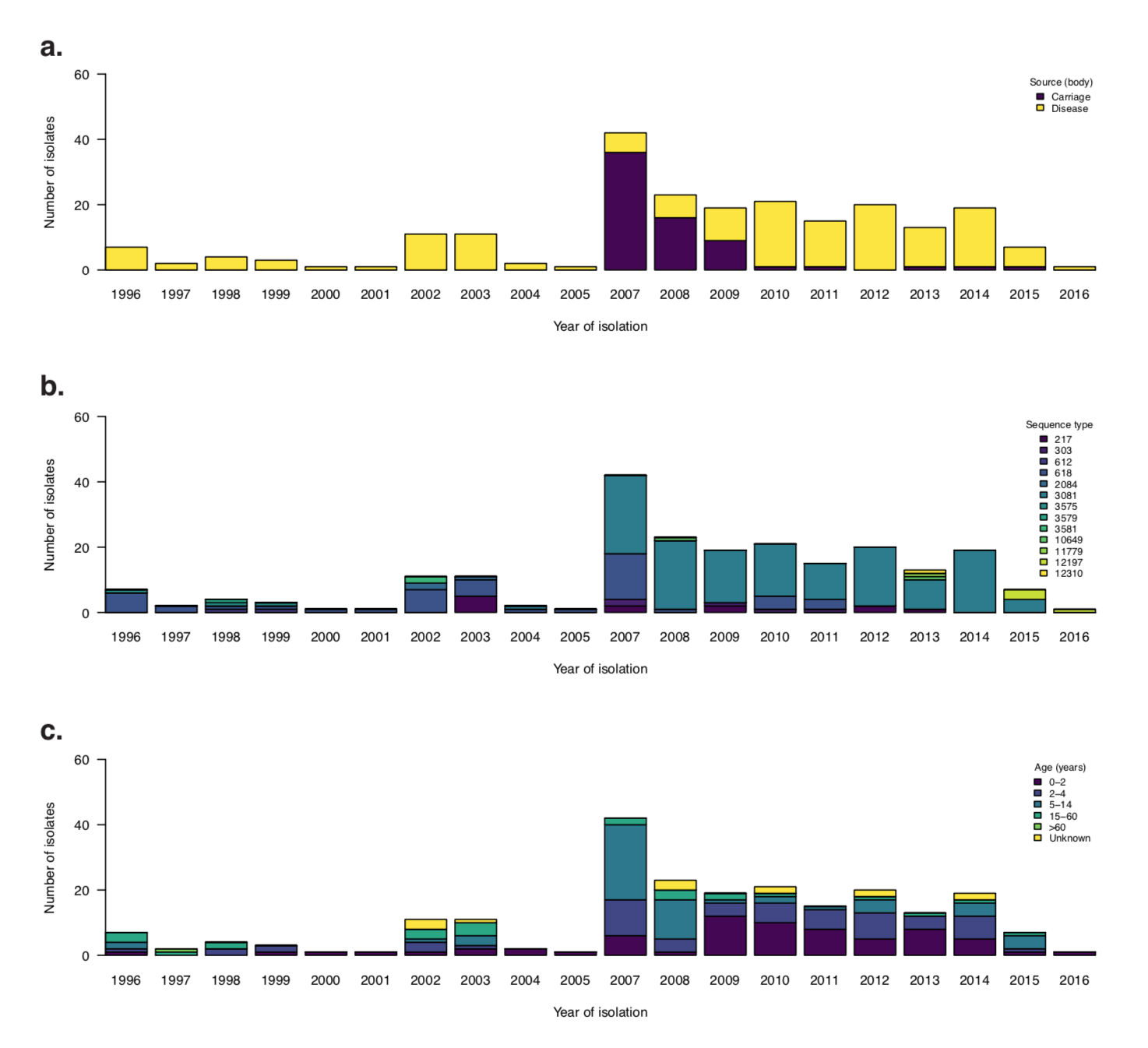


**Supplementary Fig. 1. Temporal distribution of the *S. pneumoniae* serotype 1 isolates**. The top, middle and bottom bar plots showing the number of isolates per year available for this study coloured by body isolation source, the number of isolates per year coloured by sequence type defined by multilocus sequence typing (MLST) and the number of isolates per year available coloured by the individuals’ age in years.


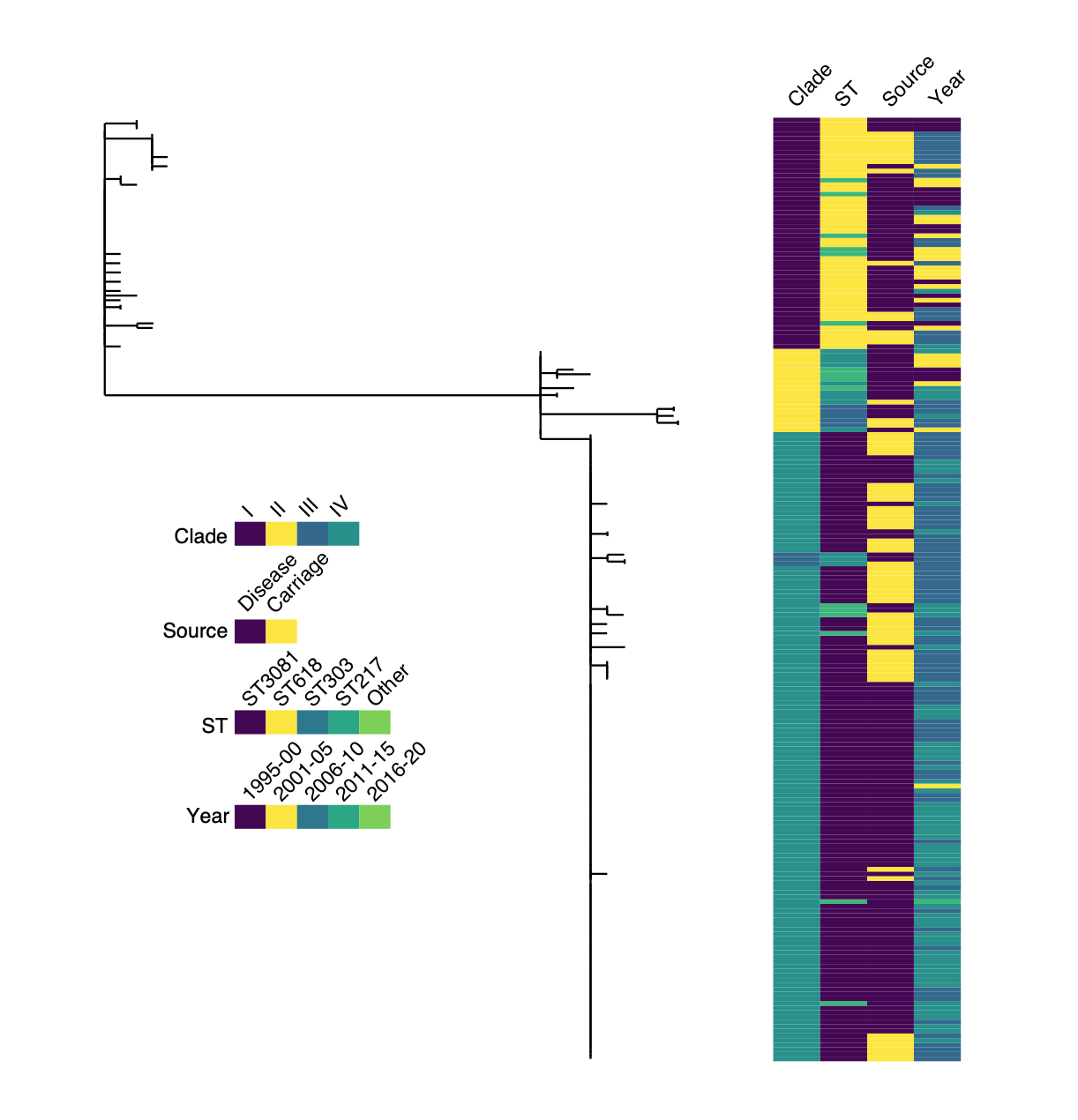


**Supplementary Fig. 2. Maximum likelihood phylogenetic tree of the serotype 1 capsule biosynthesis locus region**.


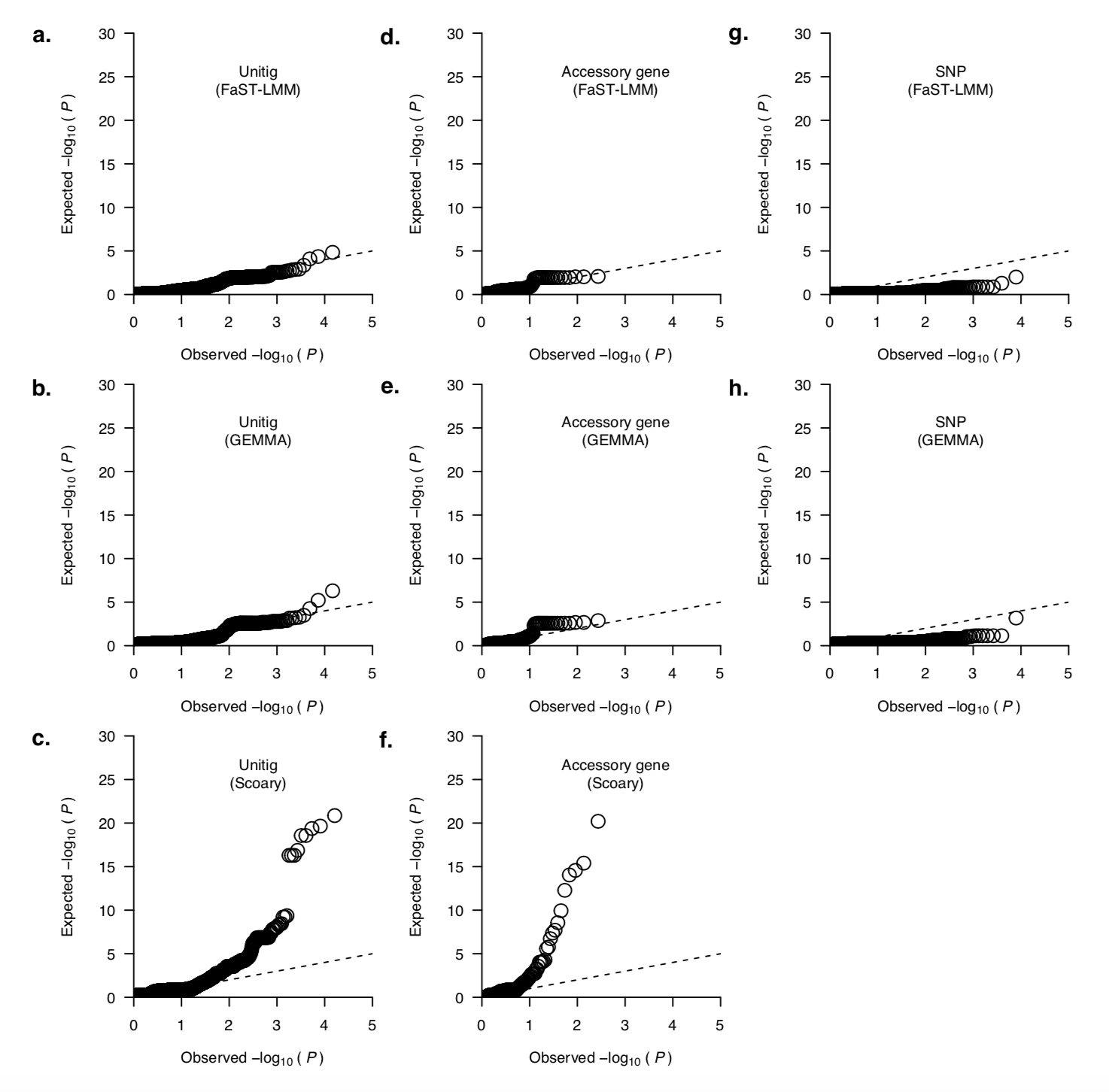


**Supplementary Fig. 3. Quantile-quantile (QQ) plots showing the expected and observed *P*-values from the GWAS of carriage- and disease-associated serotype 1 *S. pneumoniae***. The observed and expected *P*-values from the linear mixed model GWAS conducted based on the (a-c) unitig sequences, (d-f), accessory genes, and (g-h) SNPs.


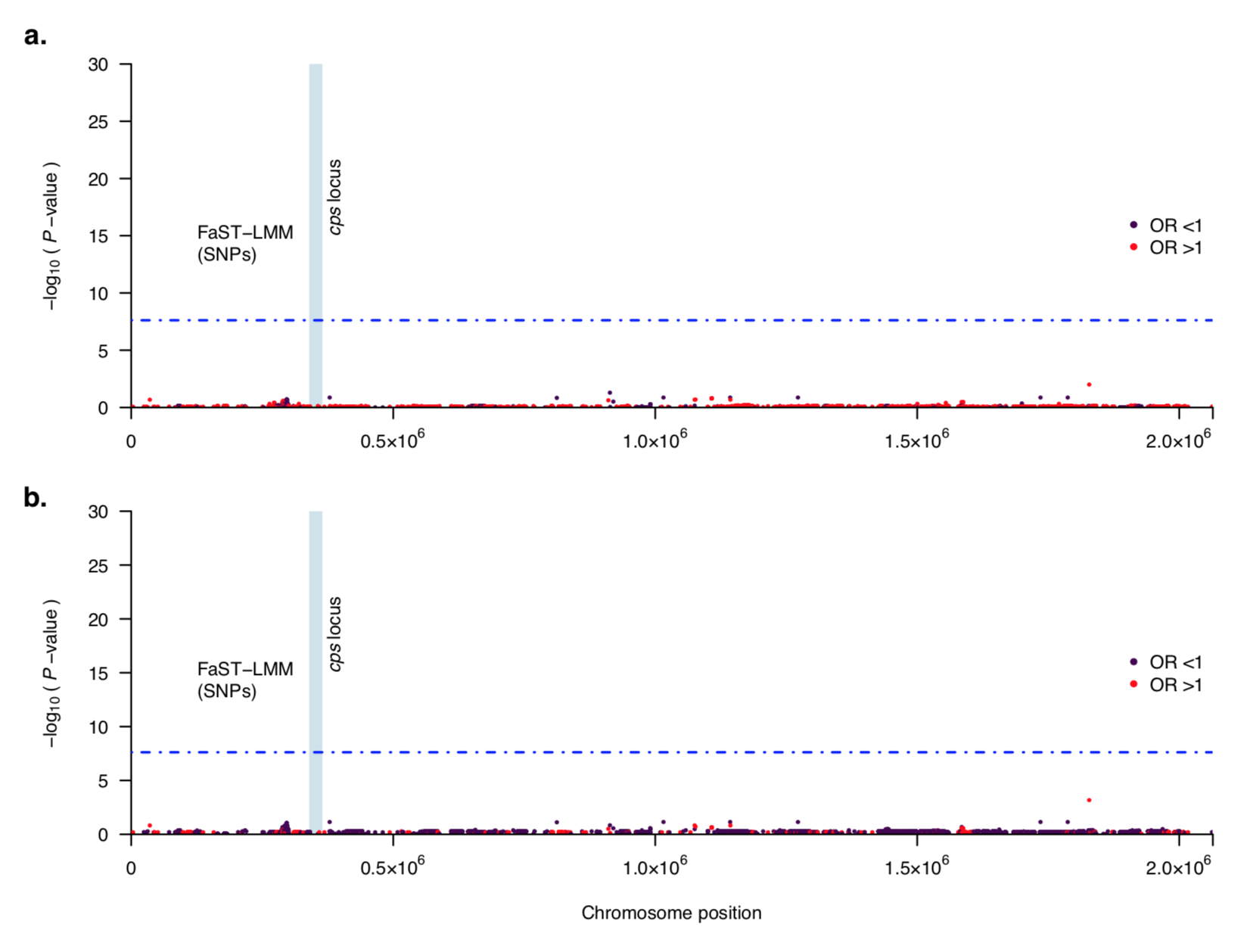


**Supplementary Fig. 4. Overview of the SNPs statistically associated with disease status in the linear mixed model GWAS**. (a) Manhattan plots showing relationship between statistical significance (-log_10_[unadjusted *P-*value]) and chromosomal location of the unitig sequences for the GWAS using (a) FaST-LMM and (b) GEMMA. The points in all the graphs are coloured based on the odds ratio as shown in the key on the right of each diagram. The blue line represents the genome-wide statistical significance threshold based on the Bonferroni adjustment. The genomic locations are based on the complete reference genome of serotype 1 strain PNI0373 from The Gambia belonging to sequence type ST168 (GenBank accession: CP001845).


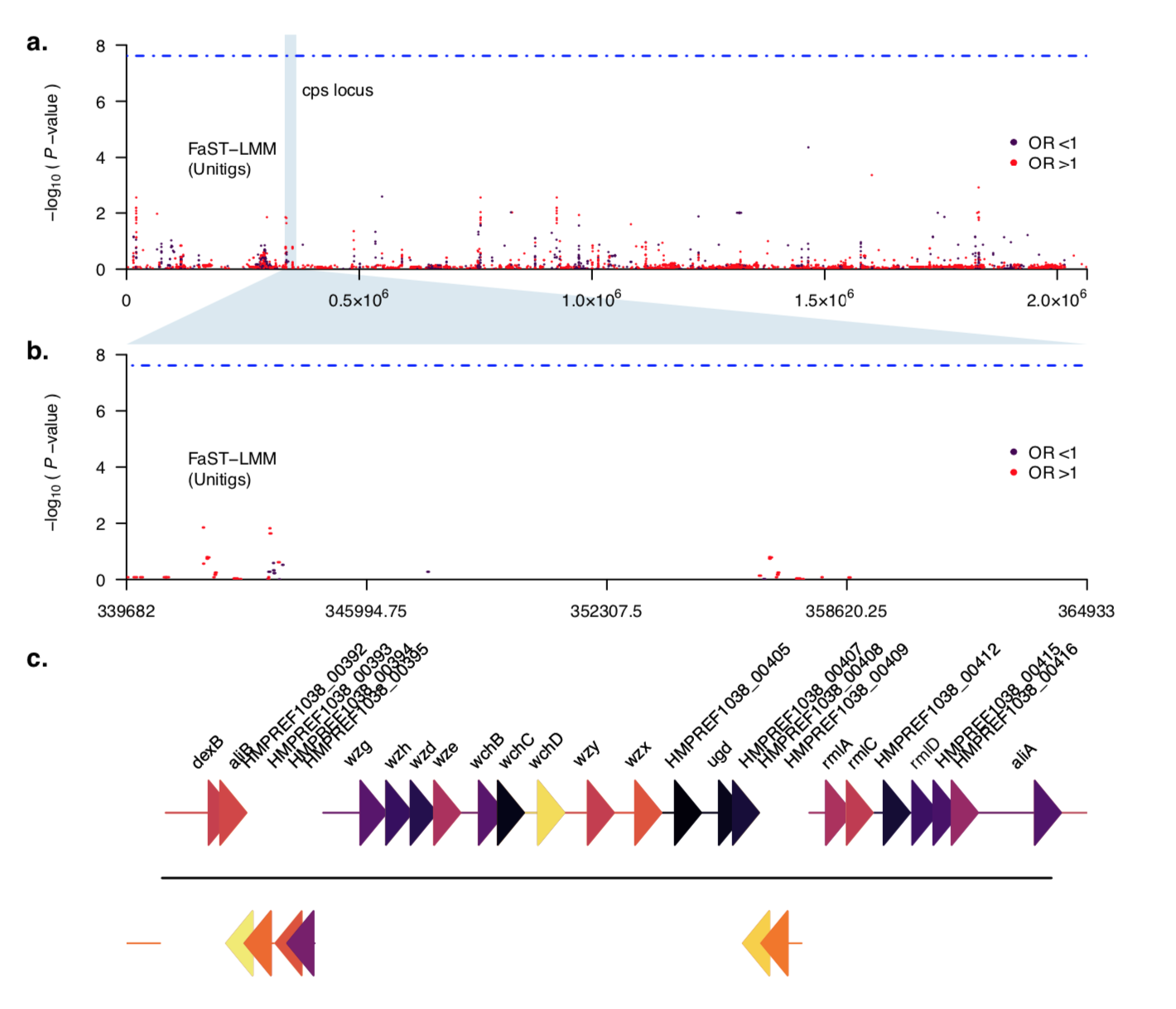


**Supplementary Fig. 5. Manhattan plot showing the unitig sequences with the capsule biosynthesis locus statistically associated with disease status**. (a) Manhattan plots showing relationship between statistical significance (-log_10_[unadjusted *P-*value]) and chromosomal location of the unitig sequences for the GWAS using FaST-LMM. (b) Zoomed Manhattan plot showing unitig sequences within the capsule biosynthesis locus. The points in all the graphs are coloured based on the odds ratio as shown in the key on the right of each diagram. The blue line represents the genome-wide statistical significance threshold based on the Bonferroni adjustment. (c) Annotation of the capsule biosynthesis locus showing genes in the forward and negative strands. The genomic locations are based on the complete reference genome of serotype 1 strain PNI0373 from The Gambia belonging to sequence type ST168 (GenBank accession: CP001845).
